# Effectiveness of Sandals Combined with Physical Therapy Versus Physical Therapy Alone on Pain and Function in Plantar Fasciitis: A Randomised Controlled Trial

**DOI:** 10.21315/mjms-01-2025-018

**Published:** 2025-08-30

**Authors:** Thanwarat Lunsarn, Torkamol Hunsawong, Kanok-On Thanasootr, Raoyrin Chanavirut, Uraiwan Chatchawan, Yodchai Boonprakop

**Affiliations:** 1 School of Physical Therapy, Faculty of Associated Medical Sciences, Khon Kaen University, Thailand; 2 The Research Centre in Back, Neck, Other Joint Pain and Human Performance (BNOJPH), Khon Kaen University, Thailand

**Keywords:** plantar fasciitis, foot orthosis, rehabilitation, exercise

## Abstract

**Background:**

Recent evidence suggests that appropriate footwear can facilitate recovery in individuals with plantar fasciitis. This study evaluated the effectiveness of combining arch-supportive, shock-absorbing flip-flop sandals with physical therapy (PT) compared to PT alone in treating plantar fasciitis.

**Methods:**

Sixty-six participants aged 20–60 years with normal body mass index and plantar fasciitis (median symptom duration = 6 months) were randomised into either an intervention group (*n* = 33; PT plus sandals) or a control group (*n* = 33; PT alone). Both groups received eight weeks of PT and home exercises. Outcome measures were assessed at baseline, four weeks, and eight weeks and included pain-related parameters, pressure pain threshold, the Foot Function Index, and the Lower Extremity Functional Scale. The global rating of change was evaluated at weeks four and eight.

**Results:**

Both groups demonstrated significant improvements across all outcome measures (*P* < 0.001). A significant time × group interaction was observed for first-step post-inactivity pain (*F* = 3.238, *P* = 0.042), with the intervention group showing greater improvement (mean reduction: 3.71 points, *d* = 2.01) compared to controls (mean reduction: 2.82 points, *d* = 1.30). Improvements in all other outcome measures were comparable between the groups.

**Conclusion:**

While PT effectively improves plantar fasciitis symptoms, adding sandals provided significantly greater improvement in first-step post-inactivity pain. Further large-scale trials with extended follow-up are warranted to confirm these findings and assess long-term efficacy.

## Introduction

Plantar fasciitis affects approximately 10% of the general population during their lifetime, predominantly among adults aged 40–60 years, particularly those engaged in occupations requiring prolonged standing or runners (1–3). This condition represents a degenerative process rather than inflammation, characterised by microtears, myxoid changes, and fascia fragmentation (4, 5). Clinically, it presents as sharp heel pain upon initial weight-bearing following periods of rest, particularly experienced as first-step morning pain (1, 4). If left inadequately managed, symptoms may become chronic and substantially impair daily functioning, occupational performance, and overall quality of life (1, 4, 6, 7). Current treatment protocols typically integrate physical therapy (PT) interventions, rest, and orthotic support, with best practice guidelines advocating for their integration to optimise therapeutic outcomes (7).

Recent meta-analyses and previous studies have demonstrated that footwear interventions offer modest yet statistically significant reductions in pain associated with plantar fasciitis (8–13). Current therapeutic footwear frequently incorporates ethylene vinyl acetate (EVA) foam and thermoplastic elastomer (TPE), materials selected for their optimal cushioning and durability (11, 13, 14). However, conventional orthotic solutions are often limited by practical constraints, including high cost, the need for professional customisation, and challenges posed by climatic conditions, particularly in tropical regions, where humidity may reduce comfort and affect adherence. Arch-supportive flip-flop sandals represent a promising therapeutic option in such climates, offering pressure-redistributing arch designs, flexible cushioning materials, enhanced ventilation that may improve treatment adherence, and seamless functional usability across both rest and activity phases.

Findings regarding the effectiveness of supportive flip-flop sandals remain mixed. While some studies have reported improvements in foot pain and function with the use of arch-supportive flip-flop sandals (15–17), others have yielded inconclusive results. One study involving predominantly overweight or obese participants found that custom insole-adapted flip-flops alleviated morning pain and improved foot function, yet had no significant impact on end-of-day pain or overall functional capacity (18). Another investigation reported minimal differences between custom-made and sham insole-adapted flip-flop sandals (19). Notably, many of these studies did not specify participants’ body mass index (BMI) categories. Furthermore, there is a paucity of research evaluating the efficacy of arch-supportive flip-flop sandals as adjuncts to PT interventions.

Several factors may contribute to these mixed findings. Higher BMI is a well-established risk factor for plantar fasciitis, with overweight/obesity showing the strongest association in non-athletic populations (20). Given this known association, most footwear intervention studies include predominantly overweight participants (mean BMI 28–32 kg/m^2^) or analyse heterogeneous BMI groups without stratification (9–13, 15–18). This population heterogeneity may mask treatment effects and contribute to inconsistent results. Recent biomechanical studies have demonstrated that overweight adults exhibit not only higher plantar pressures but also reduced gait complexity and altered sensorimotor control patterns compared to normal BMI individuals (21), suggesting that treatment responses can vary significantly between BMI groups (21, 22).

Although the condition also affects normal BMI individuals, treatment efficacy in this population remains poorly understood due to the research focus on overweight/obese participants. Focusing specifically on normal BMI participants allows the assessment of intervention effects without overweight-related biomechanical confounders, establishing clear evidence of treatment efficacy in this population before extending to more complex groups.

Since PT is an established treatment and open footwear is commonly worn in tropical climates, combining readily available arch-supportive flip-flop sandals with PT warrants clinical investigation. Therefore, this study aimed to compare the effectiveness of arch-supportive sandals combined with PT versus PT alone in normal BMI individuals with plantar fasciitis. This investigation focuses on pain-related outcomes and functional improvements to evaluate the therapeutic potential of supportive footwear as an adjunct to PT, establishing evidence for optimal combined treatment protocols in this well-defined population before extending to more complex patient groups.

## Methods

A single-blind, randomised controlled trial was conducted from August 2022 to March 2024 at the local University. The University Ethics Committee in Human Research approved the study protocol (ethical approval number HE652128), and all participants provided written informed consent before participation.

### Participants

Eligible participants were adults aged 20–60 years with unilateral or bilateral heel pain persisting for at least 4 weeks. To minimise the confounding effects of varying BMI on plantar loading and treatment response interpretation, BMI was restricted to 18.5–24.9 kg/m^2^. Key inclusion criteria were pain during initial morning steps or following periods of inactivity, minimum morning heel pain of 3/10 points on a Visual Analogue Scale (VAS), and tenderness on plantar fascia palpation (11, 16). Participants with a history of surgical intervention or fractures involving the spine, legs, ankle, or feet, or who had received corticosteroid injections for plantar fasciitis within six months, were excluded (9). Withdrawal was mandatory if pain persisted after rest or increased by two or more points from baseline.

**Figure 1:**
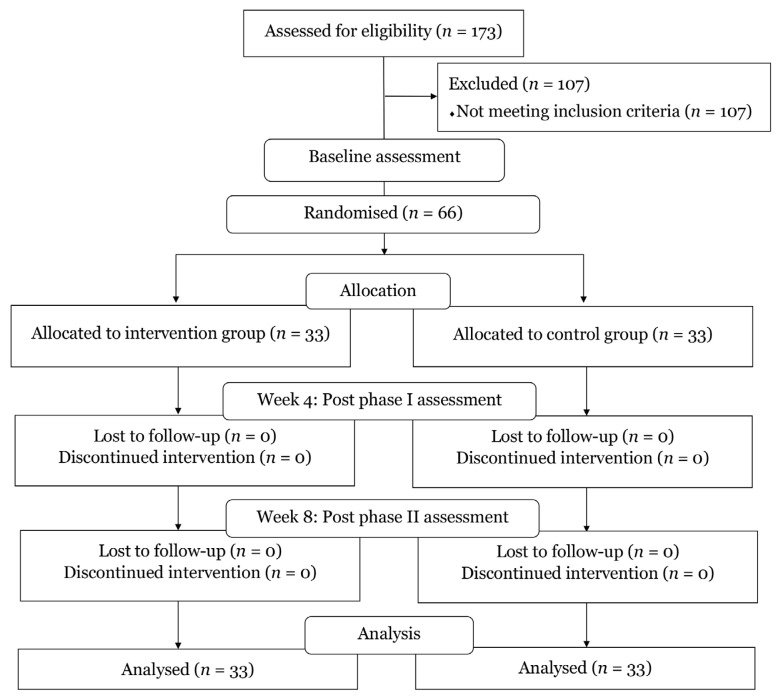
The study flow chart

The sample size was calculated using a formula for analysis of covariance (23). Based on a power of 80%, an alpha level of 0.05, and a mean difference in a first-step morning pain score of 19 mm (VAS) (24), the calculation indicated a requirement of 33 participants per group after accounting for a 10% dropout rate. Of the 173 individuals assessed for eligibility, 107 were excluded (68 for BMI above the limit, two for BMI below the limit, and 37 for other reasons), and 66 were enrolled (Figure 1).

### Research Protocol

Baseline assessments were conducted by an assessor who was a physical therapist with over five years of clinical experience, before group allocation. Following the baseline assessment, the participants were randomly allocated to groups. All follow-up assessments were performed by the same assessor who remained blinded to group allocation throughout the study period. The assessment protocol included a data collection form capturing demographic information, medical history, and pain characteristics. The self-rated and clinical measures were the Thai version of the Foot Function Index (FFI-Th), the Modified Thai Lower Extremity Functional Scale (Modified Thai LEFS), the Windlass Test for plantar fascia integrity assessment (1, 4), the Foot Posture Index-6 (FPI-6) for foot type classification (25), and a pressure pain threshold (PPT) measurement at the most painful point of the plantar fascia.

Stratified block randomisation was implemented using a computer-generated sequence to allocate participants to the intervention (Group A) or control (Group B) groups. The stratification considered three factors: intervention type, gender (male/female), and age range (20–39/40–60 years). A research assistant who was uninvolved in the recruitment and intervention processes prepared sequentially numbered, opaque, sealed envelopes using a computerised randomisation system (sealedenvelope.com) and performed the group allocation of participants. All outcome measures, including global rating of change (GRC), were reassessed at weeks four and eight post-initial intervention.

### Intervention and Control

The eight-week intervention protocol consisted of two distinct phases.

#### Phase I (Weeks 1–4)

Both groups received twice-weekly 30-min PT sessions delivered by an experienced therapist. Sessions included therapeutic ultrasound (3 MHz, continuous mode, 0.5–1.00 W/cm^2^, 5 min), subtalar joint mobilisation (grade III lateral transverse gliding, 30 repetitions), and stretching exercises (plantar fascia: 3–5 min; gastrosoleus: 30-sec sustained, two repetitions).

**Figure 2:**
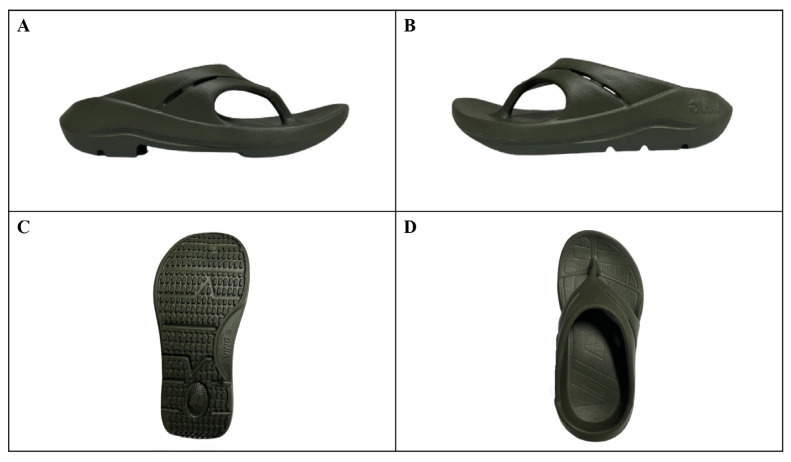
Arch-supportive flip-flop sandals (V-ING INTERTRADE CO., LTD, Thailand); A) medial view, B) lateral view, C) bottom view, and D) top view.

Both groups performed the home-based exercises throughout the study period (three sessions thrice weekly), consisting of the same mobilisation and stretching exercises taught in the clinic. The intervention group wore a commercially available arch-supportive flip-flop sandal (VING-Vari 2022, V-ING INTERTRADE CO., LTD., Thailand) selected based on its standardised manufacturing process and compliance with industrial standards. The participants were provided with new sandals at the beginning of the study and instructed to wear them for a minimum of four cumulative hours daily during weight-bearing activities (18). The four-hour minimum daily requirement ensured adequate intervention exposure across all participants while maintaining practical feasibility for daily compliance (18, 19). Daily wear time was monitored through participant self-reports using a provided wear-time log. The sandals used in this study were from the same production batch to ensure consistency in material properties. The sandal was manufactured from an EVA and TPE blend, with an insole designed for arch support and strategic weight distribution between the heel and forefoot areas (Figure 2).

#### Phase II (Weeks 5–8)

Clinic-based interventions were discontinued, while home exercises continued. The intervention group maintained their prescribed sandal-wearing protocol, while controls continued usual footwear.

For adherence monitoring, a comprehensive approach was implemented. All participants were provided with structured exercise logbooks to record their home-based exercise frequency, duration, and repetitions. Participants in the intervention group additionally documented daily sandal-wearing duration, with a minimum target of four hours per day.

Weekly telephone follow-ups were conducted by a research assistant who systematically reviewed the logbook entries with the participants, verified exercise and sandal-wearing compliance, and documented any adverse events. To validate compliance beyond self-reporting, the same research assistant conducted objective measures, including physical inspection of sandals for wear patterns and participant demonstration of prescribed exercises, to verify proper technique and protocol understanding. These assessments were conducted during routine treatment sessions and follow-up visits to ensure consistent monitoring throughout the study period.

### Outcome Measurements

#### VAS

Pain intensity was evaluated using the VAS for three conditions: first-step morning pain, first-step post-inactivity pain (first-step pain after prolonged inactivity), and average 24-hour pain. Participants marked their pain level on a 10 cm horizontal line ranging from 0 (no pain) to 10 (worst imaginable pain). A change of two or more points was considered clinically significant (24).

#### PPT

PPT was measured using a digital pressure algometer with a 1 cm^2^ rubber tip (1.12 cm diameter). Measurements were taken at the most painful tenderness point on the plantar fascia, with pressure applied perpendicular to the tissue at a constant rate of 1 kg/sec (26, 27). Participants were instructed to indicate the precise moment when the pressure sensation became painful. Three measurements were taken with 30-sec rest intervals, and the average of these measurements was used for the data analysis. Prior to data collection, intra-rater reliability testing was performed, showing good to excellent reliability (ICC3,1 = 0.85, 95% CI: 0.72–0.93, *P* < 0.001).

#### Functional Ability

Functional ability was assessed with self-rated questionnaires including the cross-culturally validated FFI-Th and the Modified Thai LEFS. The FFI-Th evaluates the impact of disorders on foot and ankle function across three domains: pain (9 items), disability (9 items), and activity limitation (5 items). Each item is scored on a 10-cm VAS. Total scores are normalised to a 0–100 percentage, with higher scores indicating greater impairment (28, 29). The Modified Thai LEFS determines functional disability in lower extremity disorders. It is comprised of 20 items rated on a 5-point scale ranging from 0 (extreme difficulty/unable to perform) to 4 (no difficulty) and yields a total score of 0–80. Higher scores indicate better function, and an improvement of 10 or more points was considered clinically significant (30, 31).

#### GRC

GRC was used to assess self-rated overall change at weeks 4 and 8 compared to baseline. Ratings were given on an 11-point scale ranging from −5 (very much worse) to +5 (very much better). A rating of zero indicated no change. A change of two or more points was considered clinically significant (32).

### Statistical Analysis

Statistical analyses were performed using SPSS version 28.0 (IBM Corp., Armonk, NY, USA). Data normality was assessed using the Shapiro-Wilk test. Demographic and baseline characteristics were summarised using means and standard deviations for continuous variables and frequencies and percentages for categorical variables. Between-group comparisons at baseline were conducted using independent *t*-tests for normally distributed continuous variables, Mann-Whitney U tests for non-normally distributed variables (age, BMI, duration of symptoms, and standing/walking duration), and chi-square tests for categorical variables (gender, side of plantar fasciitis, and foot type).

The sample size calculation was initially based on analysis of covariance (ANCOVA). However, preliminary analysis showed no statistically significant differences in baseline outcome measures between groups. This confirmed that the randomisation method effectively ensured equivalent baseline characteristics across groups, eliminating the need for covariate adjustment. Therefore, a two-way repeated measures analysis of variance (ANOVA) was conducted to examine two effects including the interaction effect (3 times × 2 treatment groups) and the within-group effects (times) and group effects. Post-hoc analyses with Bonferroni correction were performed when significant main or interaction effects were found. The effect size was calculated using Cohen’s d for within-group differences and partial eta squared (η^2^) for ANOVA results. Effect sizes were interpreted as follows: Cohen’s *d* values ≥ 0.80 were considered large, 0.50–0.79 moderate, and 0.20–0.49 small; η^2^ values ≥ 0.14 were considered large, 0.06–0.13 moderate, and 0.01–0.05 small (33–35).

This study contained no missing data, as all participants completed the required assessment sessions. Analyses were conducted according to intention-to-treat principles, with statistical significance set at *P* < 0.05.

## Results

### Participant Characteristics

A total of 66 participants with plantar fasciitis completed the study, with equal allocation to intervention (*n* = 33) and control (*n* = 33) groups. The participants in both groups were predominantly female (intervention: 78.8%; control: 81.8%). Mean age was comparable between groups (intervention: 41.45 ± 10.91 years; control: 41.64 ± 10.76 years), with median ages of 43.00 years (interquartile range [IQR]: 32.00–51.00) and 42.00 years (IQR: 36.00–51.00) for intervention and control groups, respectively. Participants had a normal range BMI (intervention: 23.06 ± 1.81 kg/m^2^; control: 22.25 ± 1.90 kg/m^2^). The median duration of symptoms was 6.00 months in both groups (intervention IQR: 3.00–18.00; control IQR: 2.50–12.00). Daily standing/walking duration was slightly higher in the intervention group (5.58 ± 2.65 h) compared to the control group (4.62 ± 2.48 h). More than half of the participants (54.5%) in both groups presented with bilateral plantar fasciitis. The FPI-6 scores were similar between groups (intervention: 5.15 ± 3.36; control: 3.91 ± 3.30). Regarding foot type, the intervention group comprised 45.45% normal, 48.48% flat, and 6.07% high-arched feet, whereas the corresponding percentage for the control group showed 54.54%, 36.36%, and 9.10%. There were no significant differences in any baseline demographic or clinical characteristics between groups (all *P* > 0.05) (Table 1).

According to the logbook records, participants in the intervention group wore sandals for an average of 5.33 ± 1.90 h per day. All participants in both groups performed their prescribed home-based exercises (100% compliance), and no adverse events were reported during the study period.

### Outcomes Measures

#### First-Step Morning Pain

Both groups demonstrated significant improvements in first-step morning pain over the 8-week period (*F* = 134.204, *P* < 0.001, η^2^ = 0.677). The intervention group showed substantial reduction from baseline (5.23 ± 2.34) to week 4 (2.36 ± 2.06) and week 8 (1.59 ± 1.84), with large effect sizes (*d* = 1.42 and *d* = 1.83, respectively). Similarly, the control group improved from baseline (5.23 ± 2.55) to week 4 (2.03 ± 2.05) and week 8 (1.71 ± 1.61), with large effect sizes (*d* = 1.45 and *d* = 1.71). No significant between-group differences were observed (*F* = 0.027, *P* = 0.871) (Tables 2 and 3).

#### First-Step Post-inactivity Pain

First-step post-inactivity pain was the only outcome showing a statistically significant time × group interaction (*F* = 3.238, *P* = 0.042), indicating differential treatment effects between groups over time. The intervention group demonstrated greater improvement from baseline (5.26 ± 1.71) to week 8 (1.55 ± 1.93), with a mean reduction of 3.71 points (95% CI: −4.56 to −2.86, *d* = 2.01). The control group also showed significant improvement but with a smaller magnitude of change (baseline: 4.62 ± 2.38; week 8: 1.80 ± 1.92; mean reduction: 2.82 points, 95% CI: −3.83 to −1.82, *d* = 1.30) (Tables 2 and 3).

#### Average 24-hour Pain

Average 24-hour pain showed significant improvement over time (*F* = 137.162, *P* < 0.001, η^2^ = 0.682) in both groups. The intervention group improved from 5.06 ± 1.54 at baseline to 1.75 ± 1.76 at week 8 (mean change: −3.31 points, 95% CI: −4.15 to −2.47, *d* = 2.02), while the control group improved from 4.72 ± 1.90 to 1.82 ± 1.64 (mean change: −2.89 points, 95% CI: −3.64 to −2.15, *d* = 1.60). No significant between-group differences were found (*P* = 0.936) (Tables 2 and 3).

#### PPTs

PPTs improved significantly over time (*F* = 20.182, *P* < 0.001, η^2^ = 0.240). The intervention group showed greater improvement from baseline to week 8 (+1.11 kg/cm^2^, 95% CI: 0.49 to 1.72, *d* = 1.05) compared to the control group (+0.69 kg/cm^2^, 95% CI: 0.11 to 1.26, *d* = 0.59) (Tables 2 and 3).

#### Functional Outcomes

The Foot Function Index showed significant improvements over time (*F* = 132.993, *P* < 0.001, η^2^ = 0.675). The intervention group improved by 30.31% (95% CI: −36.84 to −23.78, *d* = 1.95) and the control group by 27.75% (95% CI: −35.72 to −19.78, *d* = 1.75) from baseline to week 8. The Lower Extremity Functional Scale also demonstrated significant improvements (*F* = 53.337, *P* < 0.001, η^2^ = 0.455), with similar gains in both groups (intervention: +15.12 points, *d* = 1.15; control: +13.88 points, *d* = 1.08) (Tables 2 and 3).

#### GRC

Both groups reported similar levels of perceived improvement at week 8, with scores of 3.73 ± 1.18 in the intervention group and 3.64 ± 1.14 in the control group (Table 2). These scores indicate moderate to large improvements in both groups, consistent with the observed changes in other outcome measures.

## Discussion

This study investigated whether commercially available arch-supportive sandals incorporating EVA and TPE materials could enhance PT outcomes in plantar fasciitis management. Both interventions demonstrated significant improvements across all outcome measures, with a notable finding of significant time × group interaction for first-step post-inactivity pain. These findings align with recent evidence demonstrating meaningful benefits of footwear for plantar heel pain (8–13, 15–18).

Both protocols yielded substantial improvements in first-step morning pain, with similar magnitudes of change throughout the 8-week period. Both groups showed large effect sizes in pain reduction (intervention: *d* = 1.83; control: *d* = 1.71), consistent with Ribeiro et al.’s (10) findings on combined orthotic and therapeutic interventions. Average 24-h pain showed comparable improvements between groups, with both achieving clinically meaningful reductions (intervention: −3.31 points, *d* = 2.02; control: −2.89 points, *d* = 1.60), suggesting both approaches effectively address ongoing pain symptoms. These results align with previous studies showing benefits of supportive flip-flops. Costa et al. (18) demonstrated that custom insoles in flip-flops provided superior outcomes over 12 weeks, with significant between-group differences in morning pain reduction (mean difference: −1.82 cm, 95% CI: −3.3 to −0.3, *P* = 0.016). Chuter et al. (15) showed flip-flops with moulded foot-beds significantly improved pain and function compared to flat sandals. Amoako-Tawiah et al. (17) demonstrated that combined orthotics and orthotic sandals provided greater pain reduction (median change: 6 points) compared to orthotics alone (4 points, *P* = 0.003). However, these findings contrast with Fagundes et al. (19), who found no clinically significant differences between custom and sham insoles in flip-flops.

The differential improvement in first-step post-inactivity pain represents a key finding, as this common symptom of plantar fasciitis substantially impacts quality of life and functional ability. Both groups demonstrated substantial improvement from baseline to week 8, with the intervention group showing a mean reduction of 3.71 points (*d* = 2.01) and the control group showing a mean reduction of 2.82 points (*d* = 1.30), both representing large effect sizes. However, the significant time × group interaction (*P* = 0.042) indicates that the intervention group demonstrated a superior pattern of improvement over the 8-week period, suggesting that supportive sandals accelerate recovery.

This finding is particularly clinically relevant as first-step post-inactivity pain is typically challenging to treat and directly affects physical function during activity transitions. The present result aligns with Xu et al.’s (11) findings, suggesting that appropriate orthotic support can facilitate better tissue adaptation during activity transitions. The superior improvement pattern in the intervention group indicates that supportive sandals enhance patients’ ability to engage in activities requiring intermittent movement throughout the day, addressing a core functional limitation in plantar fasciitis patients.

The PPT results further supported this pattern, with the intervention group showing larger improvement (*d* = 1.05) compared to controls (*d* = 0.59). Notably, the intervention group demonstrated progressive improvement throughout the study period, while the control group showed a delayed response pattern. This difference in tissue response to mechanical loading aligns with Bishop et al.’s findings (9), suggesting that consistent arch support enhances tissue adaptation and pain modulation mechanisms.

Despite using different footwear between groups, we found no significant between-group differences in improving first-step morning pain. This may be because morning pain primarily reflects tissue response after prolonged unloaded periods, where the influence of daytime supportive footwear might be limited. The slightly higher daily standing/walking duration in the intervention group (5.58 ± 2.65 vs 4.62 ± 2.48 h) did not appear to negatively impact outcomes, suggesting that the supportive sandals may help maintain therapeutic benefits even with increased weight-bearing activities.

Functional outcomes showed substantial improvements in both groups. The Foot Function Index improved by 30.31% (*d* = 1.95) in the intervention group and 27.75% (*d* = 1.75) in controls, exceeding the minimal clinically important difference. Similarly, the Lower Extremity Functional Scale showed clinically meaningful gains in both groups (intervention: 15.12 points, *d* = 1.15; control: 13.88 points, *d* = 1.08). Although the between-group differences were not statistically significant, the consistently larger effect sizes in the intervention group suggest potential added benefits of supportive sandals, particularly in managing activity-related symptoms. These results parallel studies showing therapeutic footwear matched PT effectiveness (13). The GRC scores further supported these findings, with both groups reporting moderate to large perceived improvements (intervention: 3.73 ± 1.18; control: 3.64 ± 1.14).

The therapeutic benefits likely stem from addressing the underlying biomechanical overuse condition (4, 5). When combined with PT, this biomechanical support helps maintain proper foot mechanics during daily activities, creating an environment conducive to tissue healing. The arch-supportive sandals may provide several biomechanical advantages: reducing excessive pronation during stance to decrease tensile forces on the plantar fascia, optimising the windlass mechanism for enhanced energy efficiency, and improving plantar pressure distribution. The benefits observed in post-inactivity pain and PPTs align with mechanisms described by Thong-On and Harutaichun (36), where appropriate medial arch support reduces excessive foot pronation, affecting the entire lower extremity chain and reducing perifascial oedema. Additionally, the viscoelastic properties of EVA and TPE materials provide shock absorption, potentially reducing microtrauma to fascial insertion, particularly during heel strike when impact forces are highest.

Our study population characteristics provide important context for interpreting these results. The higher proportion of bilateral cases (54.5%) and varied foot types in our participants suggest these findings may be applicable across different structural presentations of plantar fasciitis. The average daily sandal wear time of 5.33 ± 1.90 h indicates good compliance, supporting the validity of our findings.

These findings have important clinical implications, particularly in tropical climates where traditional orthotic solutions may be impractical (7). Commercially available arch-supportive sandals offer several advantages: ready accessibility, greater affordability than custom orthoses, and immediate intervention options for clinicians and patients. Although our study did not formally evaluate cost-effectiveness, readily available supportive sandals generally represent a more economical option than custom orthoses based on market pricing. The positive outcomes in managing first-step post-inactivity pain suggest these sandals could serve as practical interventions for patients requiring frequent transitions between rest and activity periods.

Although our study demonstrates supportive sandal benefits in tropical climates, applicability varies across environmental contexts. In tropical and subtropical regions, where open-toed footwear is culturally acceptable year-round, supportive sandals may be an ideal long-term solution. However, temperate or colder climates require transitioning to enclosed supportive footwear during cooler months, raising concerns about treatment continuity and whether the biomechanical benefits persist when alternating between footwear types. In addition, the cultural acceptability of various footwear styles in professional and social settings also warrants consideration when recommending long-term management solutions for plantar fasciitis.

Several limitations should be considered when interpreting these findings. First, the 8-week duration may be insufficient to capture long-term outcomes, particularly tissue remodelling and sustained functional changes. Second, our sample was limited to individuals with normal BMI (mean: 22.25–23.06 kg/m^2^), potentially affecting generalisability to overweight or obese populations who commonly present with plantar fasciitis. Third, participant blinding to footwear interventions was not feasible, potentially introducing placebo effects or novelty bias. Our single-blind design minimises measurement bias, and the specific first-step post-inactivity pain improvement pattern suggests mechanism-specific responses. However, the recent sham-controlled study found no differences between groups, challenging positive outcomes in studies without sham controls (19). The possibility that observed benefits partly reflect placebo responses rather than solely therapeutic effects cannot be entirely excluded.

Future research should address several key areas. Long-term follow-up studies extending beyond 8 weeks are needed to assess the sustained benefits of arch-supportive sandals combined with PT. Studies involving participants with varying BMI ranges and activity levels would enhance generalisability. Additionally, investigating specific biomechanical effects, including dynamic plantar pressure analysis during various walking speeds and surfaces to quantify pressure redistribution effects, along with 3D kinematic assessment of foot and ankle motion patterns, would help validate the proposed biomechanical mechanisms. Cost-effectiveness analyses comparing commercially available supportive sandals with custom orthotic devices would also yield valuable information for clinical decision-making. Such formal cost-effectiveness analyses would provide valuable economic data to inform clinical decision-making. Multi-centre trials across different climate zones could assess the year-round feasibility of supportive sandal interventions in varying environmental conditions.

## Conclusion

Both treatment protocols effectively improved plantar fasciitis symptoms in participants with normal BMI. Arch-supportive sandals demonstrated superior management of first-step post-inactivity pain, accelerated recovery patterns, and improved PPTs. These findings indicate that sandals augment therapeutic effects through consistent arch support during daily activities and by serving as an effective adjunct to PT. They offer climate-appropriate solutions for tropical settings while combining therapeutic benefits with the convenience of daily wear and enhanced functional outcomes during activity transitions. For optimal benefit, patients should wear arch-supportive sandals for a minimum of 4 hours daily. However, the generalisability of these findings is limited to populations with normal BMI. Future research requiring extended follow-up beyond 8 weeks and diverse BMI populations would establish broader applicability and long-term therapeutic sustainability.

## Figures and Tables

**Table 1:** Baseline demographic and clinical characteristics of participants

Characteristics	Intervention Group (*n* = 33)	Control Group (*n* = 33)	*P*-value
Gender, *n* (%)			0.757^b^
Female	26 (78.8)	27 (81.8)	
Male	7 (21.2)	6 (18.2)	

Age, years	41.45 (10.91)	41.64 (10.76)	0.990^c^
Median (IQR)	43.00 (32.00–51.00)	42.00 (36.00–51.00)	

BMI, kg/m^2^	23.06 (1.81)	22.25 (1.90)	0.105^c^
Median (IQR)	23.31 (21.78–24.65)	22.48 (20.72–23.86)	

Duration of symptoms, months	11.27 (12.84)	12.42 (14.93)	0.702^c^
Median (IQR)	6.00 (3.00–18.00)	6.00 (2.50–12.00)	

Standing/walking duration, hours/day	5.58 (2.65)	4.62 (2.48)	0.133^c^
Median (IQR)	5.00 (4.00–8.00)	4.00 (2.50–6.50)	

Side of plantar fasciitis, *n* (%)			1.000^b^
Unilateral	15 (45.5)	15 (45.5)	
Bilateral	18 (54.5)	18 (54.5)	

FPI-6, score	5.15 (3.36)	3.91 (3.30)	0.135^a^
Range	−2.00 to 10.00	−3.00 to 11.00	

Foot type, *n* (%)			0.593^b^
Normal	15 (45.45)	18 (54.54)	
Flat	16 (48.48)	12 (36.36)	
High-arched	2 (6.07)	3 (9.10)	

Values are presented as mean (standard deviation) unless otherwise indicated; IQR = interquartile range; BMI = body mass index; kg/m^2^ = kilogram per square metre; FPI-6 = Foot Posture Index-6; Statistical tests:

a Independent *t*-test,

b Chi-square test,

c Mann-Whitney U test

**Table 2:** Between-group changes over time for all outcome measures (*n* = 33 per group)

Variables	Time Point	Intervention Group	Control Group	Statistics		

				Time^1^	Group^2^	Time × Group Interaction^3^
First-step morning pain	Baseline	5.23 (2.34)	5.23 (2.55)	*F* = 134.204^***^,^a^	*F* = 0.027^ns^	*F* = 0.495^ns^
	Week 4	2.36 (2.06)	2.03 (2.05)			
	Week 8	1.59 (1.84)	1.71 (1.61)			

First-step post-inactivity pain	Baseline	5.26 (1.71)	4.62 (2.38)	*F* = 103.170^***^,^a^	*F* = 0.017^ns^	*F* = 3.238*
	Week 4	2.02 (1.73)	2.56 (2.32)			
	Week 8	1.55 (1.93)	1.80 (1.92)			

Average 24-hour pain	Baseline	5.06 (1.54)	4.72 (1.90)	*F* = 137.162^***^,^a^	*F* = 0.006^ns^	*F* = 1.550^ns^
	Week 4	2.17 (1.50)	2.53 (2.01)			
	Week 8	1.75 (1.76)	1.82 (1.64)			

PPT, kg/cm^2^	Baseline	2.43 (0.90)	2.65 (1.17)	*F* = 20.182^***^,^a^	*F* = 0.010^ns^	*F* = 1.195^ns^
	Week 4	2.97 (1.09)	2.88 (1.07)			
	Week 8	3.53 (1.15)	3.34 (1.24)			

FFI, %	Baseline	41.14 (15.97)	39.44 (18.92)	*F* = 132.993^***^,^a^	*F* = 0.001^ns^	*F* = 0.272^ns^
	Week 4	17.71 (17.06)	18.16 (16.98)			
	Week 8	10.83 (14.68)	11.69 (12.22)			

LEFS, scale 0–80	Baseline	54.97 (14.49)	54.09 (14.25)	*F* = 53.337^***^,^a^	*F* = 0.562^ns^	*F* = 0.200^ns^
	Week 4	67.58 (10.10)	64.85 (12.52)			
	Week 8	70.09 (12.36)	67.97 (10.48)			

GRC, score −5 to +5	Baseline	0	0	-	-	-
	Week 4	3.45 (1.33)	3.30 (1.33)			
	Week 8	3.73 (1.18)	3.64 (1.14)			

Values are presented as mean (standard deviation); kg/cm^2^ = kilogram per square centimetre; PPT = pressure pain threshold; FFI = Foot Function Index; LEFS = Lower Extremity Functional Scale; GRC = global rating of change; ns = not significant;

1 Time effect: *F*(2, 128) for all measures;

2 Group effect: *F*(1, 64) for all measures;

3 Time × Group interaction: *F*(2, 128) for all measures;

Statistical significance ^*^P < 0.05, ^**^< 0.01, ^***^P < 0.001; All pairwise comparisons between time points were significant (*P* < 0.001) with Bonferroni adjustment;

* First-step post-inactivity pain showed a significant time × group interaction (*P* = 0.042);

a Large effect size as partial eta squared (η^2^) values ≥ 0.14.

**Table 3:** Within-group changes over time for all outcome measures (*n* = 33 per group)

Variables	Group	Time point comparison	Mean difference (95% CI)	Effect sizes
First-step morning pain	Intervention	Week 4 – Baseline	−2.86 (−3.74 to −1.98)	1.42^***^
		Week 8 – Baseline	−3.63 (−4.55 to −2.72)	1.83^***^
		Week 8 – Week 4	−0.77 (−1.39 to −0.16)	0.39^*^
	Control	Week 4 – Baseline	−3.20 (−4.10 to −2.30)	1.45^***^
		Week 8 – Baseline	−3.52 (−4.50 to −2.54)	1.71^***^
		Week 8 – Week 4	−0.32 (−1.02 to 0.37)	0.17^ns^
First-step post-inactivity pain	Intervention	Week 4 – Baseline	−3.24 (−4.04 to −2.44)	1.89^***^
		Week 8 – Baseline	−3.71 (−4.56 to −2.86)	2.01^***^
		Week 8 – Week 4	−0.47 (−1.14 to 0.20)	0.26^ns^
	Control	Week 4 – Baseline	−2.06 (−3.01 to −1.10)	0.92^***^
		Week 8 – Baseline	−2.82 (−3.83 to −1.82)	1.30^***^
		Week 8 – Week 4	−0.76 (−1.62 to 0.09)	0.35^ns^
Average 24-hour pain	Intervention	Week 4 – Baseline	−2.89 (−3.59 to −2.19)	1.87^***^
		Week 8 – Baseline	−3.31 (−4.15 to −2.47)	2.02^***^
		Week 8 – Week 4	−0.42 (−0.99 to 0.16)	0.27^ns^
	Control	Week 4 – Baseline	−2.19 (−2.89 to −1.50)	1.15^***^
		Week 8 – Baseline	−2.89 (−3.64 to −2.15)	1.60^***^
		Week 8 – Week 4	−0.70 (−1.40 to −0.01)	0.38^*^
PPT, kg/cm^2^	Intervention	Week 4 – Baseline	0.54 (−0.03 to 1.12)	0.55^ns^
		Week 8 – Baseline	1.11 (0.49 to 1.72)	1.05^***^
		Week 8 – Week 4	0.56 (0.15 to 0.98)	0.50^**^
	Control	Week 4 – Baseline	−0.23 (−0.67 to 0.22)	0.20^ns^
		Week 8 – Baseline	0.69 (0.11 to 1.26)	0.59^*^
		Week 8 – Week 4	0.46 (0.11 to 0.81)	0.41^**^
FFI, %	Intervention	Week 4 – Baseline	−23.43 (−29.71 to −17.16)	1.42^***^
		Week 8 – Baseline	−30.31 (−36.84 to −23.78)	1.95^***^
		Week 8 – Week 4	−6.88 (−11.33 to −2.43)	0.42^**^
	Control	Week 4 – Baseline	−21.28 (−28.85 to −13.72)	1.21^***^
		Week 8 – Baseline	−27.75 (−35.72 to −19.78)	1.75^***^
		Week 8 – Week 4	−6.47 (−13.09 to 0.15)	0.43^ns^
LEFS, score 0 to 80	Intervention	Week 4 – Baseline	12.61 (5.89 to 19.32)	1.02^***^
		Week 8 – Baseline	15.12 (8.26 to 21.98)	1.15^***^
		Week 8 – Week 4	2.52 (−1.94 to 6.97)	0.22^ns^
	Control	Week 4 – Baseline	10.76 (6.09 to 15.42)	0.89^***^
		Week 8 – Baseline	13.88 (8.92 to 18.83)	1.08^***^
		Week 8 – Week 4	3.12 (−0.25 to 6.50)	0.27^ns^

kg/cm^2^ = kilogram per square centimetre; CI = confidence interval; PPT = pressure pain threshold; FFI = Foot Function Index; LEFS = Lower Extremity Functional Scale; ns = not significant; Values adjusted using Bonferroni correction for multiple comparisons; Statistical significance ^*^P < 0.05, ^**^< 0.01, ^***^P < 0.001;

Effect sizes of Cohen’s *d*: large (≥ 0.80), moderate (0.50–0.79), small (0.20–0.49)
